# Case Report: Free fibula flap for post-oncologic mandibular reconstruction, first experience in Tirana

**DOI:** 10.3389/fonc.2025.1678310

**Published:** 2025-09-29

**Authors:** Alice Marzi Manfroni, Marjon Sako, Nela Mataj, Deniada Zhupa, Francesco Laganà

**Affiliations:** ^1^ Maxillofacial Surgery Unit, Istituto di Ricovero e Cura a Carattere Scientifico (IRCCS) Ospedale Policlinico San Martino, Genoa, Italy; ^2^ Maxillofacial Surgery Unit, University Hospital Center Mother Theresa, Tirana, Albania; ^3^ Private Practice Maxillofacial Surgeon, Tirana, Albania

**Keywords:** oral squamous cell carcinoma (OSCC), fibula free flap, mandibular reconstruciton, oncologic reconstruction, developing health care systems, microsurgery

## Abstract

Mandibular reconstruction using an osteocutaneous free fibular flap is a highly advanced microsurgical procedure, rarely performed in developing healthcare systems due to technical complexity and infrastructure demands. We present the first successful case performed in Tirana, Albania, demonstrating the feasibility of implementing such techniques in emerging surgical settings. A 54-year-old male diagnosed with squamous cell carcinoma of the left mandibular alveolar crest (cT4N1M0) underwent left hemimandibulectomy, bilateral neck dissection and immediate mandibular reconstruction using a right osteocutaneous fibular free flap. Preoperative Doppler ultrasonography confirmed sufficient vascular supply, excluding contraindications such as peripheral vascular disease and venous insufficiency. Fibula was harvested without tourniquet, improving vascular visualization and reducing ischemic time. A 4 × 7 cm skin paddle was included for intraoral soft tissue reconstruction. Cervical recipient vessels were isolated for microvascular anastomosis. Postoperative management involved intensive monitoring by a multidisciplinary team. Flap viability was assessed through color, temperature, capillary refill, and Doppler signals, with early mobilization and speech/swallowing therapy initiated promptly. The patient experienced an uneventful recovery with complete flap survival and functional restoration. This case highlights the potential to achieve complex microvascular reconstructions successfully in resource-limited environments through careful planning and interdisciplinary collaboration.

## 1 Introduction

Mandibular bone defects resulting from trauma, malignancy, infection, or congenital anomalies represent significant reconstructive challenges that profoundly impact patients’ quality of life and aesthetic outcomes. While contemporary techniques including prosthetic reconstruction and distraction osteogenesis have expanded treatment options, vascularized bone grafting remains fundamental for managing complex defects and promoting osseous healing (1–5).

The evolution of microvascular free tissue transfer has revolutionized maxillofacial reconstruction over the past five decades. Initial developments in free fibula transfer were subsequently refined through lateral surgical techniques and osteomyocutaneous fibular flaps. The description of osteotomy techniques enabling anatomical contouring transformed the fibula into the current gold standard for head and neck reconstruction (6–8). The vascularized fibular free flap offers unique advantages, providing immediate mechanical support while maintaining capacity for adaptive remodeling (9). Clinical indications include bone defects exceeding 6 cm, failed conventional bone grafting, infected nonunion, and post-tumor resection reconstruction, particularly when adjuvant radiation therapy is planned. The peroneal artery provides the primary vascular pedicle extending up to 15 cm when harvesting the distal fibula, supporting skin paddles up to 200 cm². Fibular reconstruction enables subsequent implant-supported prosthodontic rehabilitation, with favorable outcomes (10–12). This approach demonstrates efficacy in complex cases requiring extensive anatomical restoration, where precise three-dimensional positioning is paramount for both functional and aesthetic outcomes (9, 13–15).

Free fibular flap reconstruction represents a highly sophisticated microsurgical procedure requiring advanced technical expertise and specialized infrastructure. Beyond the technical complexity, potential complications including flap loss, vascular thrombosis, infection, donor site morbidity, and osteosynthesis failure must be effectively managed (16). These multifaceted complications contribute to the limited utilization of such reconstructive procedures in developing healthcare systems where access to specialized training, microsurgical expertise, and postoperative monitoring capabilities may be restricted. Therefore, we present our institutional experience with a free fibular flap reconstruction case, demonstrating feasibility of implementing these procedures in developing healthcare environments.

## 2 Case presentation

**Figure 1:**
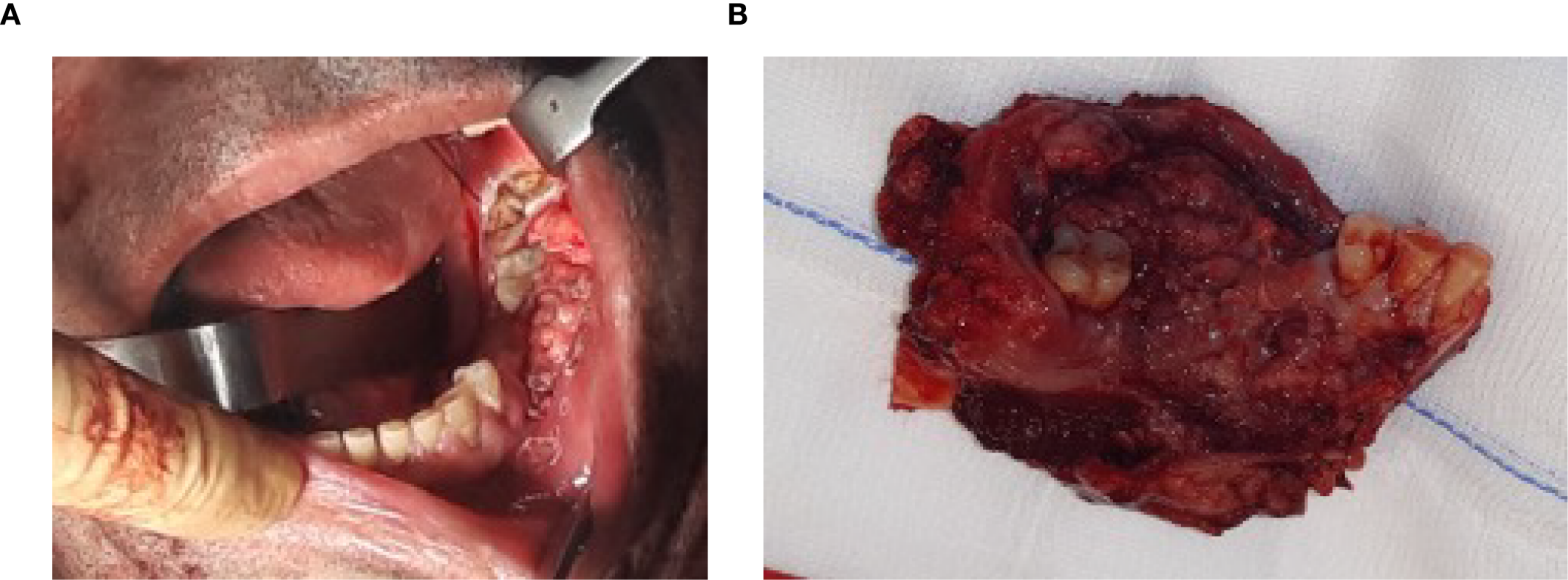
**(A)** Oral squamous cell carcinoma involving the left mandibular gingiva and bone, extending from the retromolar trigone region posteriorly to tooth 3.4 anteriorly. **(B)** Hemimandibulectomy showing the resected neoplasm, with the mandibular bone of the third quadrant, associated dental elements 3.2, 3.3, 3.4, and 3.8, and surrounding soft tissues.

A 54-year-old Caucasian male presented with an asymptomatic voluminous (**Figure 1A**), partially exophytic, ulcerated, and bleeding mass of the left mandibular alveolar crest. The lesion was neither spontaneously painful nor tender to palpation, with no reported hypoesthesia of the fifth cranial nerve. Palpation of the cervical lymph nodes revealed one lymphadenomegaly at the ipsilateral level IIb. In the medical history, the patient reported tobacco consumption (7.5 pack/years) and occasional alcohol use. Given the clinical presentation, diagnostic workup was indicated including contrast-enhanced computed tomography of the head, neck and thorax, along with histopathological examination of the neoplasm trough incisional biopsy. The prescribed diagnostic investigations revealed a squamous cell carcinoma of the left mandibular gingiva with partial infiltration of the underlying bone and a contrast-enhancing lymph node at the left level IIb lateral cervical station, which was staged as cT4N1M0.

Following multidisciplinary consultation with radiologists, oncologists, and radiation oncologists, the case was deemed suitable for radical surgical treatment. The most appropriate ablative procedure was planned, consisting of left hemimandibulectomy to maintain oncologically safe margins of at least 1cm from the macroscopically visible neoplasm, modified radical neck dissection of the ipsilateral neck, and, if suspicious lymph nodes were present, supraomohyoid neck dissection of the contralateral neck. The most appropriate reconstructive option appeared to be the contralateral fibular osteocutaneous flap, whose osseous component would enable reconstruction of the resected hemimandible while permitting future implant-supported prosthetic rehabilitation, and whose vascular pedicle length would provide greater flexibility in the depleted neck. The patient met inclusion criteria with adequate lower extremity vascularity confirmed by Doppler ultrasonography demonstrating strong independent pulses at both dorsalis pedis and posterior tibial arteries. Exclusion criteria including peripheral vascular disease, hypoplastic anterior tibial artery, venous insufficiency, and deep vein thrombosis were ruled out through preoperative assessment. Written informed consent was obtained from the patient for the surgical intervention and for publication of data and images.

After prophylactic antibiotic administration, the procedure was performed under general anesthesia by a single surgical team over a twelve-hour period. Patient positioning ensured efficient workflow and adequate microscope access for microvascular anastomosis. The decision was made to operate without tourniquet to maintain better vascular visualization and reduce bleeding during fibular harvesting, accepting increased harvesting time in exchange for reduced ischemic time and preservation of the microsurgical phases.

Following temporary tracheostomy, a cervical incision extending from the left to right mastoid process and continuing over the left sternocleidomastoid muscle was created. Bilateral neck dissections were performed, followed by a left hemimandibulectomy extended from dental element 3.1 to the ipsilateral mandibular angle, maintaining oncologically safe resection margins of 1cm from the macroscopically visible tumor (**Figure 1B**), therefore completing the ablative phase. Appropriate recipient cervical vessels were identified and isolated for subsequent vascular anastomosis.

**Figure 2:**
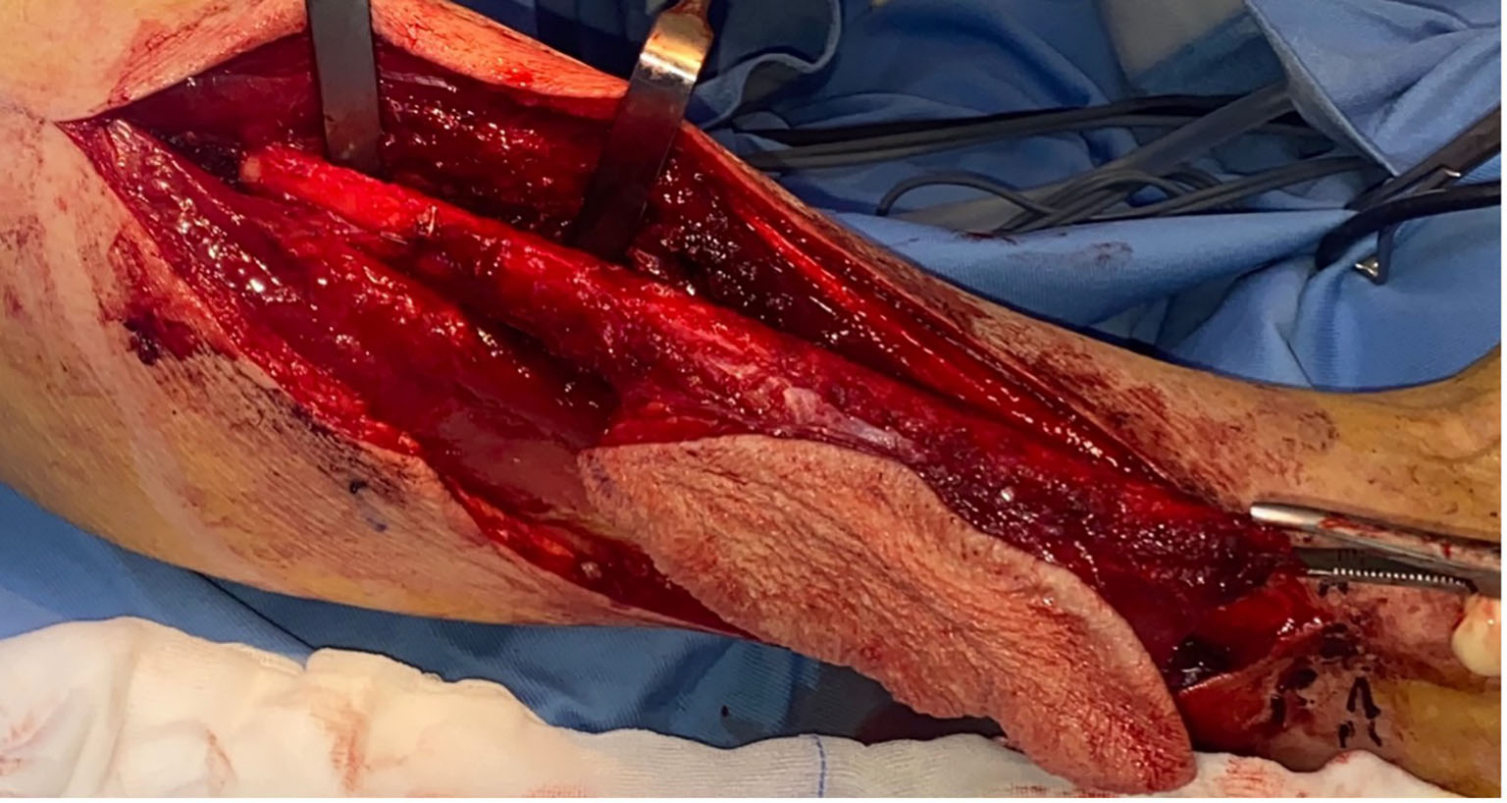
Right osteocutaneous fibula flap harvesting, with cutaneous paddle supplied by two perforating vessels, utilized for intraoral soft tissue reconstruction. The use of the contralateral fibula allows for a longer pedicle length by enabling its posterior exit, thereby reducing the cervical trajectory required to reach the donor vessels.

The right fibula was harvested using the lateral surgical approach with a longitudinal incision along the lateral lower leg, preserving 7 cm of bone at both extremities to maintain peroneal nerve function proximally and ankle joint stability distally. A skin paddle measuring 4 × 7 cm was designed at the distal aspect of the fibular flap for intraoral soft tissue reconstruction (**Figure 2**). Care was taken to avoid injury to the peroneal artery and its two accompanying venae comitantes during dissection.

**Figure 3:**
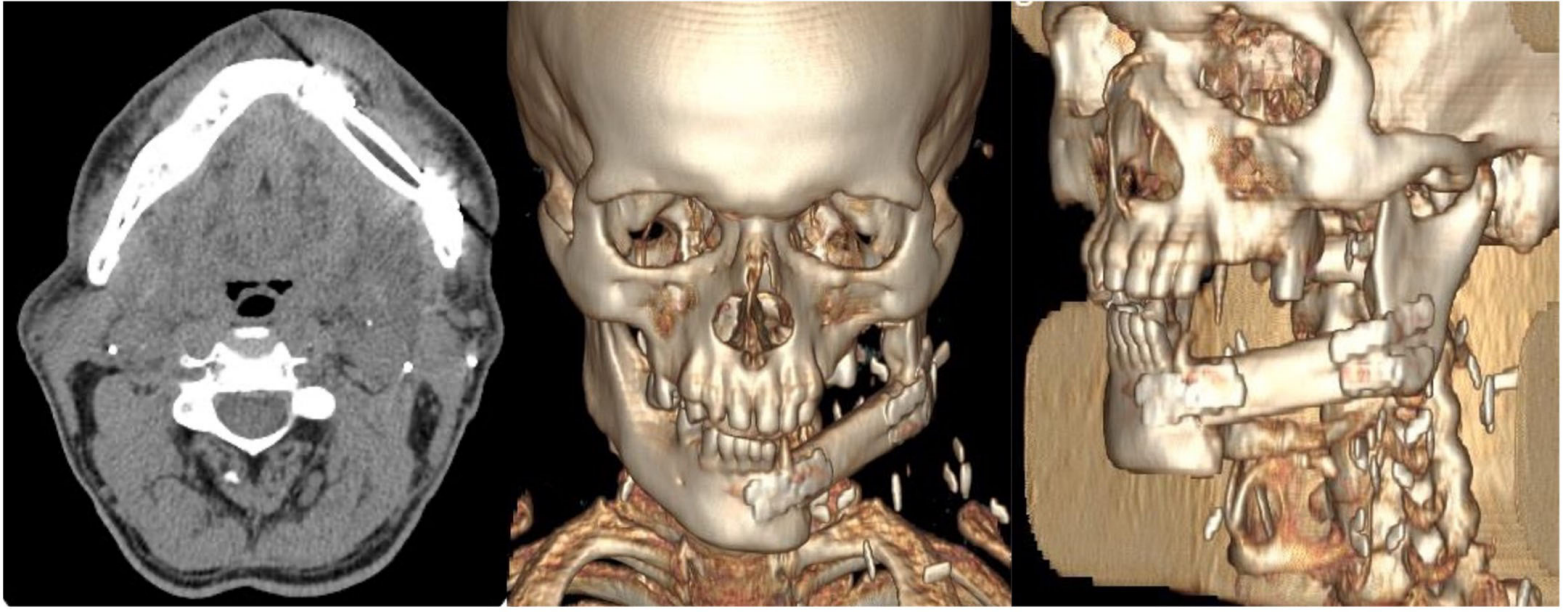
Post-operative CT scans of the patient showing correct placement of the free fibula flap to reconstruct the left hemimandibular defect. The flap is secured with miniplates to the residual mandibular segments and positioned superiorly at the level of the alveolar crest to ensure optimal future implant rehabilitation.

Following pedicle identification and division, the fibular flap underwent bench preparation to remove excess bone and lengthen the vascular pedicle while preserving periosteum. The fibula was positioned as a single segment extending from the symphysis to the left mandibular angle and secured with four 2.0 titanium compression miniplates and two screws per side (**Figure 3**).

Microsurgical reconstruction was performed under operative microscopy with end-to-end anastomosis between the peroneal artery and facial artery, and end-to-end venous anastomosis between the dominant pedicle vein and the Farabeuf venous trunk. Flap viability was confirmed through assessment of skin paddle perfusion, capillary refill, temperature, and Doppler signals. Two closed-suction drains were placed, and layered closure of the neck and intraoral skin paddle was completed. The fibular donor site was closed primarily, and the skin paddle defect was reconstructed with a split-thickness skin graft.

Postoperative care involved intensive monitoring by a multidisciplinary team including intensivists, surgeons, physical therapists, and speech/swallowing therapists. As this represented the inaugural case of this surgical procedure, the patient underwent continuous monitoring in the intensive care unit for the initial three postoperative days, with fluid balance assessment through intake and urinary output measurement via bladder catheterization, along with continuous evaluation of blood pressure and oxygen saturation parameters. Throughout the surgical procedure, systolic arterial pressure was maintained below 100mmHg, while continuous blood pressure monitoring was performed during the initial 72 hours, avoiding systolic values exceeding 130mmHg by administering for hypertension. Laboratory examinations were performed on postoperative days 1, 3, 7, and 13. Attentive nursing care focused on flap monitoring during the critical first 72 hours when anastomosed vessels undergo re-epithelialization. Flap assessment included evaluation of color, temperature, capillary refill, bleeding response to lancet prick, and Doppler signals, with particular attention to venous congestion signs which account for 80-90% of flap failures. During hospitalization, the patient received broad-spectrum antibiotic therapy throughout the admission, analgesic and gastroprotective treatment, and deep vein thrombosis prophylaxis. Enteral nutrition was administered via nasogastric tube for the initial 10 days, followed by transition to semi-liquid oral feeding. The tracheostomy cannula was removed 7 days postoperatively. Physical therapy was initiated early to prevent perioperative complications including deep vein thrombosis and pulmonary embolism. Speech and swallowing therapy with nutritional support was implemented to maintain caloric intake during recovery. The patient demonstrated successful healing without complications, maintaining flap viability throughout the postoperative period with appropriate functional restoration, and was discharged after a 14-days hospital stay.

The definitive histopathological report confirmed oral squamous cell carcinoma with 7 ipsilateral lymph node metastases without extranodal extension, yielding a pathological staging of pT4aN2b. Following multidisciplinary discussion, the patient commenced adjuvant radiotherapy beginning at the fifth postoperative week (**Table 1**). Currently, the patient is in the fourth year of postoperative follow-up, alive and without evidence of disease recurrence. He reports overall good quality of life without major limitations in dietary intake or speech function and expresses high subjective satisfaction regarding facial morphological restoration. We awaited the resolution of radiotherapy effects before initiating implant-supported prosthetic rehabilitation planning, which the patient will commence in the upcoming months.

**Table 1:** Timeline of diagnostic and therapeutic management pathway.

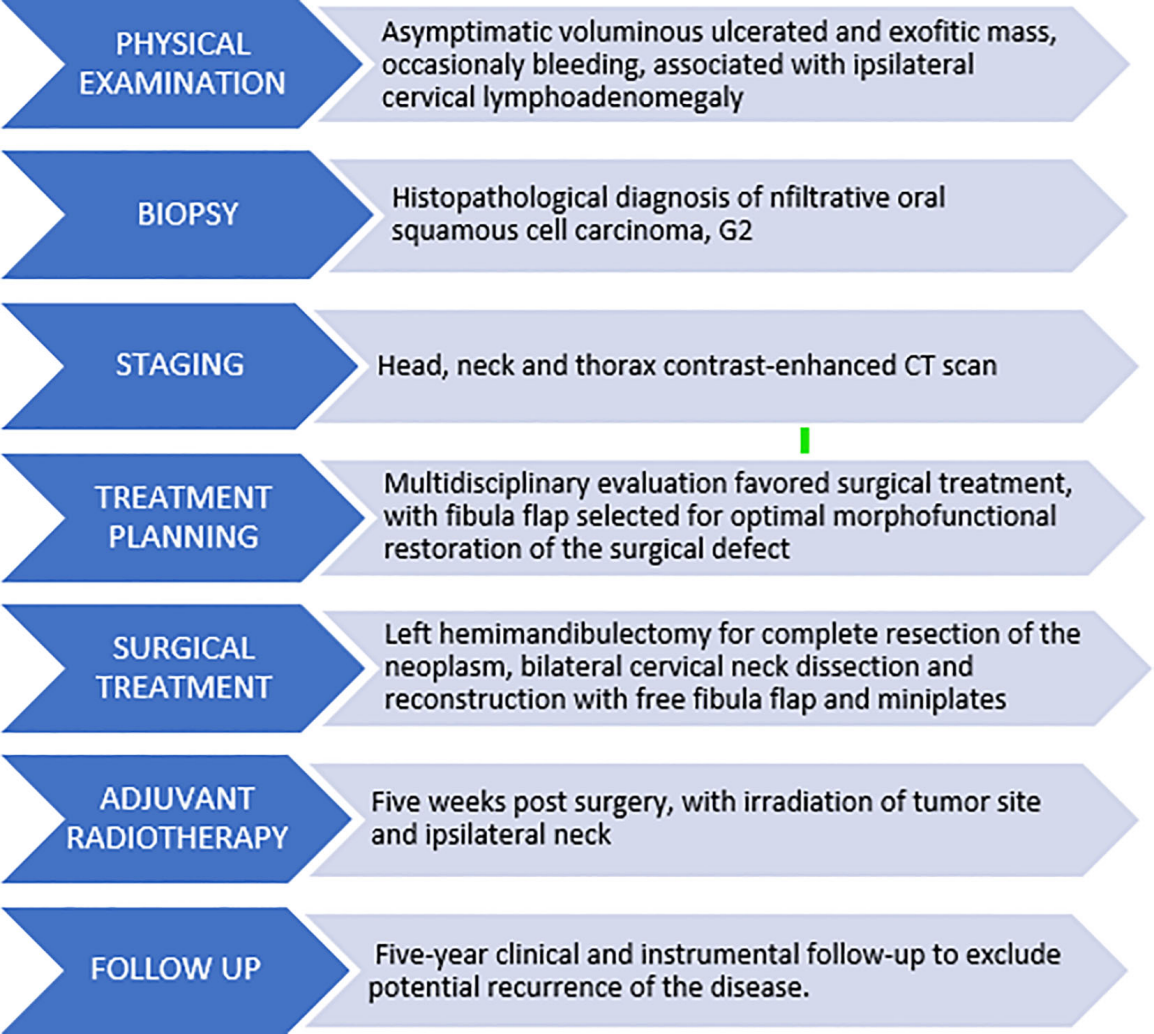

## 3 Patient perspective

I first noticed the lesion when a small ulcer appeared in my mouth and did not heal. Instead, it gradually enlarged and sometimes bled when I brushed my teeth. Since I felt no pain, I assumed it would eventually resolve on its own. Unfortunately, it did not. When it reached a worrisome size, I sought care from the maxillofacial surgery team in Tirana, where I immediately underwent several investigations, including a biopsy and CT scan. The diagnosis of carcinoma was frightening; I was deeply worried about losing the ability to speak or eat, and about being left with a severely disfigured face. When the surgeons suggested reconstruction using the fibula, I was also concerned that I might not be able to walk normally again. Nevertheless, I understood that the disease was progressing quickly and needed to be treated. The postoperative period was far from easy, especially learning to manage the tracheostomy tube, being unable to speak or eat by mouth for several weeks, and gradually regaining the ability to walk with the help of a walker and crutches. Over time, however, things improved significantly, and by the time I returned home, I was almost independent again. Another major challenge was radiotherapy, which caused swelling, severe dryness due to lack of saliva, and further difficulties with eating. Fortunately, these side effects resolved after a few months. Today, I have returned to my normal life: I am able to speak, eat, and I feel comfortable with the appearance of my face.

## 4 Discussion

Free fibular flap reconstruction has been documented in the literature as the gold standard for mandibular reconstruction following oncologic resection, trauma, or infectious bone loss. Since Hidalgo’s seminal work establishing the technical foundations of fibular free flap mandibular reconstruction, this procedure has demonstrated consistently superior outcomes in terms of bone healing, functional restoration, and long-term stability (1). Another valid reconstructive option for mandibular defects is the free iliac crest flap, which has proven highly adequate for morphological restoration of the mandibular profile while supporting subsequent implant rehabilitation (17, 18). However, the vascular pedicle length of the fibula free flap compared to that of the free iliac crest flap provides greater confidence in anastomotic geometry and donor vessel identification with broader selection options (19), thereby making it more feasible for less experienced microsurgeons to perform in a vascularly depleted neck. An alternative osseous reconstructive option for mandibular defects is the free scapular border flap, which presents a shorter vascular pedicle compared to the fibular flap and demonstrates inferior bone quality that precludes adequate and effective implant-supported prosthetic rehabilitation (20). Conversely, the fibula has proven to be an excellent foundation for implant rehabilitation, representing a particularly advantageous consideration for young patients with favorable rehabilitative prospects. Contemporary studies continue to validate its efficacy in post-oncologic, traumatic and malformative surgery, with recent research demonstrating that patients undergoing free fibula flap mandibular reconstruction achieve satisfactory masticatory performance, confirming the functional benefits of this reconstructive approach (11, 12, 21–24).

Severe cases of oral cavity and head and neck malignancies often require extensive surgical excision to achieve oncologic control. However, in many developing healthcare systems, such procedures are frequently carried out without adequate reconstructive planning due to limited access to microsurgical expertise and infrastructure. As a result, patients are left with significant functional and aesthetic deficits, leading to poor quality of life, impaired speech and swallowing, nutritional complications, and increased psychological distress. Furthermore, the absence of reconstruction increases the risk of wound dehiscence, infection, chronic fistula formation, and long-term disability. Until now in Albania, reconstructive options for head and neck oncological cases were limited to either no reconstruction or reconstruction with pedicled rotational or advancement flaps such as the pectoralis major. Free bone graft reconstruction is contraindicated, particularly given the requirement for adjuvant radiotherapy initiation between the fourth and sixth postoperative weeks, as this timeframe does not allow adequate osseous graft integration and leads to reconstructive failure. For this reason, free flaps are considered, with the fibular flap being the primary choice due to its morphological adequacy and osseous component capable of supporting dental implants for younger patients who may undergo subsequent dental rehabilitation.

Despite the extensive literature supporting free fibular flap reconstruction and detailed step-by-step protocols available for surgical guidance, this sophisticated microsurgical technique remains underutilized and undocumented in developing healthcare systems. The complexity of the procedure demands advanced technical expertise, specialized equipment, and multidisciplinary infrastructure that may not be readily available in all clinical settings. The learning curve for microsurgical techniques is substantial, requiring extensive training in both harvest and recipient site preparation, vascular anastomosis, and postoperative flap monitoring protocols. Furthermore, fibular flap reconstruction carries inherent risks of complications affecting both the flap and donor site including flap necrosis, vascular thrombosis, infection, donor site ankle instability, peroneal nerve injury, and compartment syndrome (16). These potential complications may necessitate revision surgery, prolonged hospitalization, and additional specialized interventions, further emphasizing the need for comprehensive microsurgical expertise and institutional resources to manage such complex cases effectively.

The establishment of free fibular flap programs in emerging healthcare environments necessitates comprehensive training initiatives that address multiple aspects of patient care. Essential components include preoperative patient selection and vascular assessment, surgical team coordination, microsurgical technique acquisition, and intensive postoperative monitoring protocols. The multidisciplinary nature of these procedures requires collaboration between head and neck surgeons, plastic surgeons, anesthesiologists, intensivists, and specialized nursing staff, all of whom must be adequately trained in the unique requirements of microvascular reconstruction. Training programs should emphasize both theoretical knowledge and practical skills acquisition, incorporating cadaveric workshops, mentorship with experienced microsurgeons, and graduated case complexity to ensure competency development. The implementation of such programs in developing healthcare systems could significantly expand access to advanced reconstructive options for patients with complex mandibular defects, ultimately improving functional outcomes and quality of life.

The successful integration of free fibular flap reconstruction into emerging healthcare systems represents not only a technical achievement but also a paradigm shift toward comprehensive reconstructive capabilities. As demonstrated in our case, the feasibility of implementing these procedures in resource-limited environments can be achieved through dedicated training initiatives and institutional commitment to developing microsurgical expertise. This expansion of advanced reconstructive techniques to underserved populations addresses a critical gap in healthcare accessibility and represents an important step toward global standardization of optimal surgical care.

## 5 Conclusion

Free fibular flap reconstruction remains the gold standard for mandibular reconstruction due to its proven reliability, functional efficacy, and long-term success. Despite its underutilization in developing healthcare systems, this technique can be effectively implemented through structured training programs and dedicated institutional support. Our experience demonstrates that with targeted education, multidisciplinary collaboration, and investment in microsurgical infrastructure, advanced reconstructive options can be made accessible even in resource-limited settings. This represents a critical advancement toward equitable global surgical care and improved quality of life for patients with complex mandibular defects.

## Data Availability

The original contributions presented in the study are included in the article/supplementary material. Further inquiries can be directed to the corresponding author.
